# MicroRNAs and polymorphisms influencing MICA immune modulation

**DOI:** 10.1007/s11033-026-12959-5

**Published:** 2026-10-20

**Authors:** Maria Beatriz Nicoletti, Ingrid Miranda Ferraz, Amanda Cristina Moço, Erick da Cruz Castelli, Camila Ferreira Bannwart Castro

**Affiliations:** 1 Medical School, Botucatu Campus, School of Medicine, Molecular Genetics and Bioinformatics Laboratory, São Paulo State University (UNESP), Botucatu, State of São Paulo Brazil; 2 General and Applied Biology Graduate Program, São Paulo State University (UNESP), Institute of Biosciences, Botucatu, SP Brazil; 3 Pathology Graduate Program, São Paulo State University (UNESP), Institute of Biosciences, Botucatu, SP Brazil; 4 Molecular Genetics and Bioinformatics Laboratory, Experimental Research Unity UNIPEX - Av. Prof. Mário Rubens Guimarães Montenegro, s/n UNESP , Botucatu, SP 18618687 Brazil

**Keywords:** NKG2D Signaling, 3′UTR Variability, Allele-Specific Regulation, Natural Killer Cells, Stress-Induced Ligands

## Abstract

MicroRNAs (miRNAs) are small non-coding RNAs that play a central role in post-transcriptional gene regulation. They act mainly by interacting with sequences located in the 3′ untranslated regions (3′UTRs) of messenger RNAs (mRNAs), thereby influencing mRNA stability and translation. Through these mechanisms, miRNAs contribute to the fine-tuning of multiple biological pathways, including immune activation, inflammatory signaling, and cellular stress responses. Among the genes involved in immune surveillance, the major histocompatibility complex class I polypeptide-related sequence A (*MICA*) has emerged as an important target for post-transcriptional regulation due to its role as a ligand for the activating receptor NKG2D (natural killer cell group 2-D), which is expressed in natural killer (NK) cells and cytotoxic T lymphocytes. *MICA* is highly polymorphic, and several allelic variants differ in sequence composition, structural organization, and expression patterns, potentially influencing miRNA–mRNA interactions. Growing evidence suggests that miRNA-mediated regulation of MICA contributes to immune modulation in cancer, viral infections, and autoimmune disorders, particularly through mechanisms associated with immune evasion and altered NKG2D signaling. In addition, genetic polymorphisms and structural variations within regulatory regions, especially within the 3′UTR, can alter miRNA binding affinity by creating, disrupting, or modifying target sites. This mini review discusses the current understanding of miRNA-mediated regulation of MICA, emphasizing the impact of allelic diversity and regulatory polymorphisms on post-transcriptional control. Furthermore, it highlights current knowledge gaps and future perspectives regarding allele-specific regulation.

## Tiny molecules, major control: post-transcriptional regulation by microRNAs

MicroRNAs (miRNAs) are small non-coding RNA molecules, approximately 18–24 nucleotides in length, that function as post-transcriptional regulators of gene expression [1]. Since the discovery of the first miRNA, lin-4, in *Caenorhabditis elegans* [2, 3], these molecules have been extensively studied because of their central role in the modulation of several cellular processes. More than 46,000 hairpin precursor miRNAs and 74,000 mature miRNA products from 323 species are cataloged in the miRBase database (Release 23.1; accessed September 21, 2026) [4].

The miRNAs are highly conserved across species and predominantly exert repressive effects on messenger RNAs targeted by them (target mRNAs) [5]. In general, this regulation occurs through binding to the 3′ untranslated region (3′ UTR) of mRNAs, resulting in translational repression and/or transcript degradation [6]. This interaction is primarily mediated by the so-called miRNA “seed” region, located between nucleotides 2–8 at the miRNA 5′ end, although additional interactions within the 3′ region also contribute to the stability and specificity of the pairing [5].

**Fig. 1:**
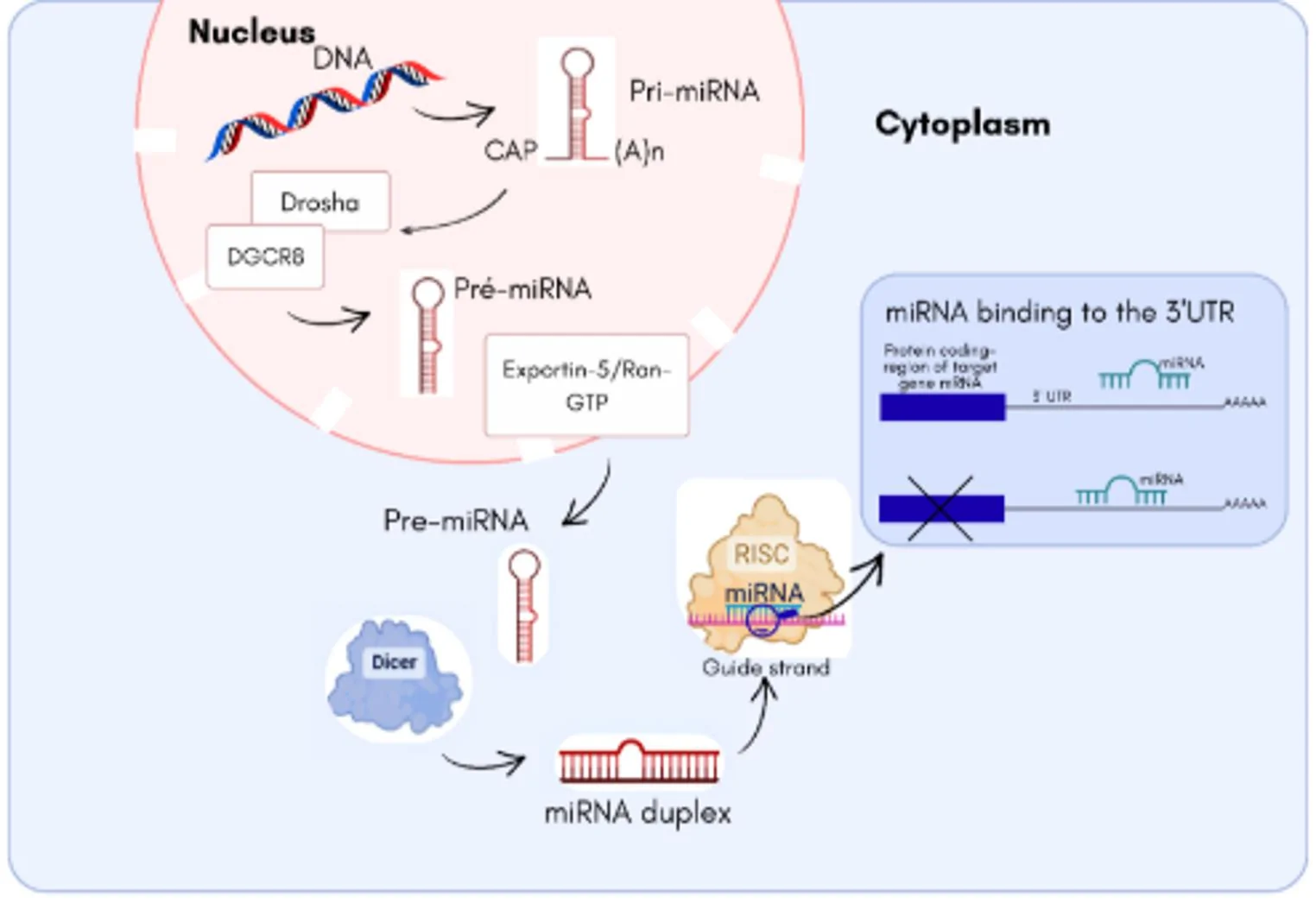
Canonical biogenesis pathway of miRNAs. miRNAs are transcribed from genomic DNA as primary transcripts (pri-miRNAs), which undergo nuclear processing by the Drosha–DGCR8 complex to generate precursor miRNAs (pre-miRNAs). These precursors are exported to the cytoplasm via Exportin-5/RanGTP and subsequently cleaved by Dicer, producing double-stranded mature miRNAs. One strand is incorporated into the RNA-induced silencing complex (RISC), guiding sequence-specific interactions with target mRNAs. Created by the authors using Canva and Microsoft PowerPoint

The biogenesis of miRNAs begins in the nucleus with the transcription of primary precursors (pri-miRNAs), which display a characteristic hairpin structure. These transcripts are processed by the RNase III enzyme Drosha, generating precursor miRNAs (pre-miRNAs), which are then exported to the cytoplasm and further cleaved by the enzyme Dicer (Fig. 1). The final product is an RNA duplex, in which one strand, known as the guide strand, is incorporated into the RNA-induced silencing complex (miRISC), while the complementary strand is degraded. The miRISC, primarily composed of the Argonaute (AGO) protein and the mature miRNA, mediates gene repression [1].

The mechanism of action of miRNAs depends on the degree of complementarity between the miRNA and its target mRNA. Extensive pairing may lead to mRNA cleavage, whereas partial interactions predominantly result in translational repression, often accompanied by deadenylation and decapping, ultimately leading to transcript degradation. Although the 3′ UTR is considered the primary site of interaction, miRNA response elements (MREs) may also be found within the coding sequence (CDS), the 5′ UTR, and, in some cases, promoter regions, suggesting that miRNAs can exert broader regulatory functions, including transcriptional modulation [7].

The 3′ UTR regions from mature transcripts play a central role in this context, acting as regulatory platforms that integrate the actions of miRNAs and RNA-binding proteins. In addition to influencing mRNA stability, localization, and translation, recent evidence suggests that these regions may also function as scaffolds for protein–protein interactions, thereby expanding their functional impact [8].

Although miRNAs are classically described as post-transcriptional repressors, their regulatory functions extend beyond direct gene silencing. Under specific conditions, such as cellular stress or nutrient deprivation, miRNAs may also contribute to gene expression activation [7, 9]. This effect can occur through indirect mechanisms, including the repression of transcriptional or post-transcriptional inhibitors, thereby relieving inhibitory constraints on downstream targets. In addition, miRNAs may participate in complex regulatory networks in which the repression of a given target triggers downstream regulatory events that ultimately result in the activation of specific genes [10, 11]. Beyond their roles in gene regulation, miRNAs have also been proposed to enhance the robustness of biological systems by modulating gene expression noise and stabilizing protein levels against stochastic fluctuations in mRNA abundance, thereby contributing to the maintenance of cellular homeostasis [12].

miRNA activity is also influenced by the subcellular dynamics of the miRISC complex. This complex can shuttle between the nucleus and cytoplasm and redistribute in response to cellular stimuli, such as stress or translational inhibition. It is frequently associated with stress granules and P-bodies, which are membrane-less cytoplasmic structures that function as sites for RNA storage, degradation, and translational repression, particularly of mRNAs [13]. Additionally, miRNAs can be exported into the extracellular environment, where they exhibit high stability and may act as signaling molecules [14].

In the immune system, miRNAs play essential roles in the fine-tuning of both innate and adaptive immune responses. They act largely as negative regulators, preventing excessive immune activation and contributing to immune homeostasis [15, 16]. Experimental evidence has shown that disruption of miRNA biogenesis, such as Dicer deletion, impairs immune cell development, including B-cell differentiation. In addition, specific miRNAs, such as miR-342, are also involved in regulating hematopoietic fate by modulating the balance between lymphopoiesis and myelopoiesis [15, 16].

Several miRNAs regulate key processes of adaptive immunity by modulating the proliferation, differentiation, and function of B and T cells [17]. Similarly, in innate immunity, miRNAs participate in regulating inflammatory pathways and host responses to pathogens. Dysregulation of these mechanisms has been associated with a variety of diseases, including autoimmune disorders such as systemic lupus erythematosus and multiple sclerosis, as well as inflammatory and hypersensitivity diseases [17]. A notable example is miR-148a, which regulates the expression of HLA-C (Human Leukocyte Antigen C) through binding to its 3′UTR. Variations affecting this interaction have been associated with altered HLA-C expression levels, influencing susceptibility to both infectious and immune-mediated diseases. Increased HLA-C expression has been linked to improved control of HIV infection, whereas dysregulation of this pathway has also been implicated in inflammatory disorders such as Crohn’s disease, highlighting the broad immunological impact of miRNA-mediated regulation [18].

Taken together, these findings highlight miRNAs as central components of gene regulation and cellular homeostasis, with direct implications in both physiological and pathological processes.

## When cells raise the alarm: MICA, a stress-induced molecule

The genes of the MHC class I chain-related (MIC) family were independently identified by Bahram et al. (1994) and Leelayuwat et al. (1994) as a novel group of genes structurally related to MHC class I molecules. Located within the human MHC region, *MICA* and *MICB* are classified as non-HLA genes and are positioned in close proximity to the HLA-B and HLA-C loci. Among the MIC family members, *MICA* exhibits the highest degree of polymorphism currently described, followed by MICB [19, 20]. Other members of the family, including MICC, MICD, MICE, MICF, and MICG, were later described; however, they were classified as pseudogenes with no known function, meaning that only *MICA* and *MICB* are considered functionally active [21].

The MHC is located on chromosome 6p21.3 and is one of the main genetic determinants of immune responses [22]. Within this genomic context, the *MICA* gene is located within the MHC, approximately 46.4 kb centromeric to the *HLA-B* gene, and spans about 15.5 kb [23]. According to the *IPD-IMGT/HLA database* (version 3.64, Access: May 2026), 727 *MICA* alleles encoding 281 distinct protein sequences have already been described, highlighting its remarkable genetic diversity.

MICA primary function is to act as a ligand for the activating receptor NKG2D, a member of the C-type lectin family expressed on natural killer (NK) cells, γδ T lymphocytes, and CD8 + αβ T lymphocytes [24, 25]. The interaction between MICA and NKG2D promotes the cytotoxic activation of these immune cells and contributes to the elimination of cells undergoing physiological or pathological stress [26].

**Fig. 2:**
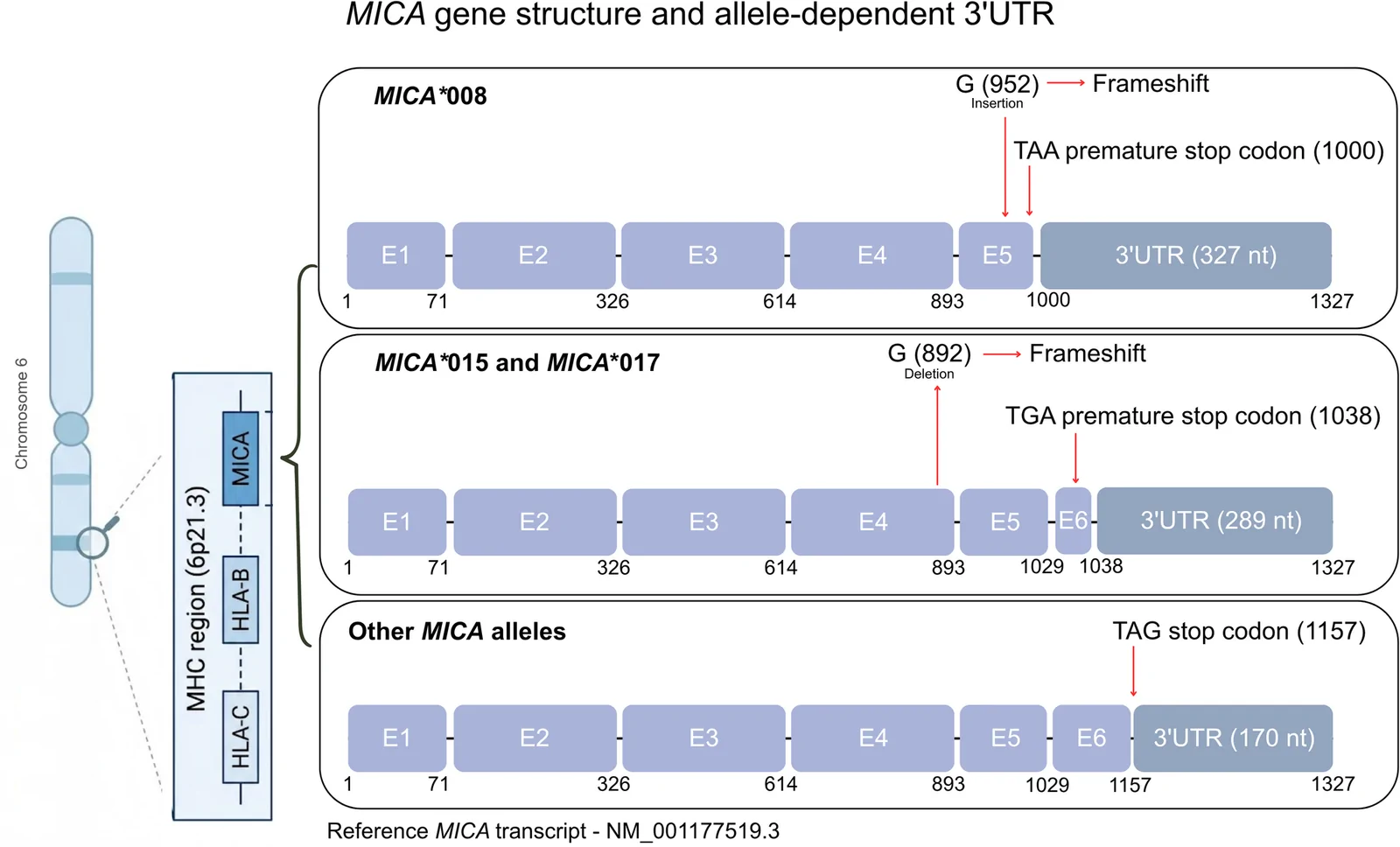
Genomic structure of the human *MICA* gene and the allele-dependent 3′UTR region. The *MICA* gene is located in the Major Histocompatibility Complex (MHC) region on chromosome 6p21.3 and consists of six exons (E1–E6) separated by intronic regions, with the reference transcript (NM_001177519.3 [51]), spanning positions 1 to 1327. Frameshift-causing insertions/deletions (indels) within exon 5 generate three distinct boundaries for the 3′UTR region. In the MICA*008 allele, a guanine (G) insertion at position 952 alters the reading frame and introduces a premature TAA stop codon at position 1000, resulting in a 327-nt 3′UTR region. In the MICA*015 and MICA*017 alleles, a guanine (G) deletion at position 892 alters the reading frame differently, introducing a TGA stop codon at position 1038 and a 289-nt 3′UTR region. Other *MICA* alleles maintain the original reading frame and utilize the canonical TAG stop codon at position 1157, resulting in the most common 3′UTR length of 170 nt. The figure does not represent all described *MICA* alleles; it serves only as a comparison among the most common ones. Created by the authors using Canva and Microsoft PowerPoint

Structurally, the *MICA* gene is composed of six exons. Exon 1 encodes the signal peptide, whereas exons 2, 3, and 4 encode the extracellular α1, α2, and α3 domains, respectively [19, 27]. Exon 5 encodes the transmembrane region and part of the cytoplasmic tail, while exon 6 encodes the final amino acids of the protein as well as the 3′UTR [19, 27] (Fig. 2). This canonical gene structure gives rise to a membrane-bound protein that is expressed on the cell surface [24, 25].

Basal MICA expression is observed in only a few cell types, including epithelial cells, endothelial cells, fibroblasts, and peripheral blood mononuclear cells [28]. However, its expression can be strongly induced under conditions of cellular stress, including malignant transformation, infection by intracellular pathogens, oxidative damage, DNA damage, and autoimmune processes [21, 24, 26, 29]. Under these conditions, membrane-bound MICA promotes immune surveillance by signaling cellular distress and triggering NKG2D-mediated activation of cytotoxic immune cells.

In addition to its membrane-bound form, MICA can also be released as a soluble molecule (sMICA). This process mainly occurs through proteolytic cleavage mediated by matrix metalloproteinases (MMPs) and members of the ADAM family (a disintegrin and metalloproteinase), particularly ADAM10, ADAM15, and ADAM17 [30, 31]. In this context, the endoplasmic reticulum protein ERp5 interacts with the α3 domain of MICA, promoting conformational changes that facilitate cleavage of the molecule [32, 33].

Unlike membrane-bound MICA, which stimulates NKG2D-mediated immune responses, soluble MICA exerts predominantly immunosuppressive effects. Upon binding to NKG2D, sMICA induces receptor internalization and lysosomal degradation, reducing NKG2D surface expression and consequently impairing the cytotoxic activity of NK cells and CD8 + T lymphocytes [34–36]. As a result, increased levels of sMICA may facilitate immune evasion by tumor and infected cells [37–39].

Beyond proteolytic shedding, genetic polymorphisms can also influence the form in which MICA is expressed. Exon 5 contains a short tandem repeat (STR) encoding a variable number of alanine residues, allowing the classification of alleles into variants such as *MICA*-A4, A5, A6, A7, A8, A9, and A10 [20, 40]. However, some alleles present a Guanine insertion within this STR (usually when there are 5 repeats in the STR), which alters the reading frame and generates a premature stop codon, resulting in a truncated protein with partial loss of the cytoplasmic tail [41–44]. This polymorphism is known as A5.1.

The truncated protein derived from this variant can be released in exosomes or remain associated with the cell membrane through a glycosylphosphatidylinositol (GPI) anchor [45]. The MICA*008 allele, which is associated with this structure, is among the most frequent *MICA* alleles reported in Brazilian populations [46–48]. Consistent with this observation, MICA*008 is one of the most prevalent *MICA* alleles worldwide and has been reported as the most frequent allele in several European and non-European populations [49].

Taken together, these characteristics demonstrate that MICA functions as an important sensor of cellular stress and a critical regulator of immune activation. Its high genetic diversity, combined with the structural and functional differences among variants, reinforces the biological relevance of this gene and supports the growing interest in understanding the mechanisms that regulate its expression, including post-transcriptional modulation mediated by miRNAs.

## Many faces, many functions: polymorphism and allelic diversity of *MICA*

The high allelic diversity of *MICA* results in important structural and functional differences that can modulate its interaction with NKG2D and, consequently, the intensity of the immune response. One of the best-characterized examples is the polymorphism at position 129 (rs1051792), which defines variants with either high (Met) or low (Val) affinity for the receptor [50]. The 129-Met variant exhibits a greater capacity to activate NKG2D, whereas the 129-Val variant is associated with weaker signaling [46].

In addition, a recent study proposed a classification of functional *MICA* haplotypes into Types I and II. These haplotypes are defined by a set of six linked polymorphic residues located at positions 36, 129, 173, 206, 210, and 215. Type I molecules are characterized by the combination C36-M129-K173-G206-W210-S215, whereas Type II molecules carry the alternative residues Y36-V129-E173-S206-R210-T215. Although the methionine/valine dimorphism at position 129 is often highlighted because of its strong impact on NKG2D binding, this residue is typically inherited together with the other five polymorphic sites, and the biological effects associated with *MICA* are thought to reflect the combined influence of the entire haplotype [50].

The main functional difference between these haplotypes is their affinity for NKG2D. Type I molecules exhibit significantly higher binding affinity and can activate NK-cell signaling pathways at lower concentrations than Type II molecules. Consequently, individuals carrying the Type I phenotype may display more robust immune surveillance against tumor or infected cells. However, this heightened responsiveness may also represent a double-edged sword, potentially increasing susceptibility to autoimmune diseases when compared with the Type II phenotype, which presents a higher activation threshold [50].

Beyond alterations in the protein sequence, *MICA* polymorphism also affects expression levels and the protein’s subcellular fate. The MICA*008 allele, which is the most prevalent in several populations, is an A5.1 allele associated with a premature stop codon [51], resulting in a truncated protein anchored by GPI and frequently released into the tumor microenvironment, including through exosomes [46, 52]. In contrast, the MICA*010 allele is retained in the endoplasmic reticulum and shows reduced surface expression, thereby limiting its ability to interact with the receptor [53].

The allelic variability of *MICA* has important clinical relevance and has been associated with susceptibility to several autoimmune diseases, neoplastic conditions, and transplant-related complications. In autoimmune diseases, specific alleles appear to differentially modulate NKG2D-mediated immune activation. The MICA*010 allele, for instance, has been described as a risk factor for psoriasis and rheumatoid arthritis, whereas MICA*045 has been shown to be strongly associated with susceptibility to systemic lupus erythematosus (SLE) [53]. These associations suggest that alterations in MICA expression, stability, or interaction with the NKG2D receptor may contribute to the breakdown of immune tolerance and the maintenance of chronic inflammatory processes.

In addition to conventional allelic variants, structural deletions involving the *MICA* gene have also been described. These deletions result in the absence of *MICA* on chromosome 6, generally due to a large deletion of approximately 100 kb between the *HLA-B* and *MICB* loci [54]. Two main patterns have been identified: a complete deletion, which is in strong linkage disequilibrium with *HLA*-B*48:01 and MICB*009:01 N, and a partial deletion that removes essential regulatory regions, including the promoter and exon 1, and has been reported in association with HLA-B*41:01:01. Interestingly, the complete deletion of *MICA* is commonly associated with the null allele MICB*009:01 N, resulting in a haplotype predicted to lack functional MIC molecules encoded by that chromosome [54]. The prevalence of these null variants varies across populations, being higher in East Asians and in individuals with Native American ancestry (such as populations from Mexico and Peru), while being almost absent among Europeans. Most carriers are heterozygous and therefore retain one functional copy of the gene. Nevertheless, rare homozygous individuals carrying MICA*del and MICB*009:01 N have been reported [54]. Although these observations indicate that complete loss of MIC molecules is compatible with life, the biological consequences of this genotype remain poorly understood [54].

Beyond structural variation, polymorphisms within the *MICA* 3′UTR may alter the binding of regulatory miRNAs, thereby influencing allele-specific expression levels. This includes members of the miR-17 ~ 92 family and related paralog clusters, such as miR-20a and miR-106b, which have been reported to directly regulate *MICA* expression [51]. Variations affecting their binding sites may differentially modulate *MICA* expression among alleles, suggesting that the functional impact of *MICA* depends not only on protein structure but also on its fine regulation at the mRNA level [51].

In the oncological context, variants associated with enhanced release of soluble or exosome-associated MICA, such as MICA*008 [45], as well as variants exhibiting lower avidity for the NKG2D receptor, including those carrying valine at position 129 [55], favor mechanisms of tumor immune evasion and have been linked to the progression of malignancies such as hepatocellular carcinoma and multiple myeloma [50]. Furthermore, in transplantation settings, incompatibility between donor and recipient *MICA* alleles may induce the formation of donor-specific antibodies (DSA) against MICA, which have been associated with renal and cardiac graft rejection even in the absence of anti-HLA antibodies [53]. Taken together, these findings demonstrate that distinct *MICA* alleles exhibit different capacities for immune regulation and activation, positioning the MICA genotype as an important determinant of disease susceptibility and therapeutic response.

## MICA under control: regulation by miRNAs

MICA protein expression is also regulated by post-transcriptional mechanisms, among which miRNAs play a prominent role [56]. These small non-coding RNAs modulate MICA expression mainly through interactions with specific binding sites located in the *MICA*’s 3′UTR of the mRNA, thereby reducing MICA transcript and/or protein expression depending on the miRNA and cellular context [56–58].

The functional relevance of this post-transcriptional regulation was first demonstrated by Stern-Ginossar et al., who identified endogenous human miRNAs that target conserved sites within the 3′UTRs of *MICA* and *MICB*. These miRNAs were shown to negatively regulate MICA and MICB protein expression and to maintain their surface levels below a functional threshold under basal conditions, thereby limiting inappropriate activation of NKG2D-mediated immune responses. During acute cellular stress, this miRNA-mediated regulation facilitates the rapid upregulation of MICA and MICB at the cell surface, promoting recognition by NKG2D-expressing NK cells. Conversely, the authors found that relevant miRNAs were overexpressed in several tumor cells and that their increased expression contributed to reduced MICA/MICB surface levels and impaired NK-cell-mediated recognition, providing a mechanism by which tumors may evade immune surveillance [59].

Several cellular miRNAs have been described as direct regulators of MICA. Among them, miR-106b and other members of the miR-25/93/106b cluster have been shown to regulate MICA through its 3′UTR [60]. Similarly, miR-20a has been experimentally demonstrated to target the MICA 3′UTR and reduce MICA expression in colorectal cancer cells [61]. In addition, miR-182 has been associated with increased NK-cell cytotoxicity and Prf1 expression, potentially through indirect modulation of the NKG2D/NKG2A axis, which may influence interactions with their ligands, including MICA [62]. These findings indicate that miRNA-mediated regulation is integrated into cellular stress response networks and malignant transformation processes.

Beyond endogenous miRNAs, viruses can also encode miRNAs that modulate NKG2D ligand expression. In Epstein–Barr virus (EBV), miR-BART7 indirectly reduces MICA expression through the TGFβ1/c-Myc/MICA regulatory axis, contributing to immune evasion by impairing NK cell-mediated cytotoxicity [58, 63].

Experimental evidence demonstrates that this regulatory mechanism is directly relevant across diverse pathological contexts. In cancer, overexpression of miRNAs that target MICA is associated with reduced surface expression of this molecule, thereby impairing recognition by immune effector cells. In ovarian cancer, for example, elevated levels of miR-20a correlate with lower MICA/B expression, contributing to impaired NK-cell-mediated cytotoxicity [62, 64]. Similarly, during viral infections, viral miRNAs can repress NKG2D ligands such as MICA, thereby potentially reducing NK-cell-mediated immune recognition [58].

Therefore, miRNA-mediated regulation contributes to a fine-tuning of cellular stress responses by modulating the expression of MICA and other NKG2D ligands. Under physiological conditions, this mechanism helps prevent excessive immune activation and tissue damage by maintaining ligand expression below the threshold required for cytotoxic activation [27]. However, in pathological settings such as cancer and viral infections, altered miRNA expression may be exploited by tumor or infected cells to reduce MICA expression, promote immune evasion, and impair the activity of NK cells and cytotoxic T lymphocytes [27].

The main functional consequence of this regulation is the modulation of the MICA–NKG2D interaction. Reduced MICA expression at the cell surface can impair NKG2D-mediated recognition by NK cells and contribute to immune evasion [65]. This mechanism not only contributes to tumor progression but may also facilitate metastatic dissemination and the persistence of infections [62].

Taken together, these findings support a significant role for miRNAs in regulating MICA expression and, consequently, in modulating immune surveillance. Therefore, understanding these interactions is essential for developing therapeutic strategies to restore the expression of NKG2D ligands and enhance antitumor immune responses.

## A matter of affinity: influence of variants in the 3′UTR on miRNA–mRNA interaction

Post-transcriptional gene regulation represents a complex process in which the 3′UTRs play a central role in controlling mRNA stability, translation, and localization [66]. These regions contain cis-regulatory elements that interact with trans-acting factors, such as RNA-binding proteins (RBPs) and miRNAs, thereby dynamically modulating gene expression [66, 67].

The integrity of the 3′UTR sequence is essential for proper miRNA recognition, meaning that small nucleotide alterations, including single nucleotide polymorphisms (SNPs) and structural variants (Insertions and Deletions), can significantly modify this regulatory interaction [51, 67]. These alterations may occur through different mechanisms, including disruption of pre-existing binding sites, the creation of novel binding sites (*de novo*), or changes in the affinity between miRNAs and mRNAs [51, 67]. Changes within the sequence that binds the miRNA seed region may disrupt or substantially reduce target recognition, whereas other variants may generate new complementary sequences for miRNAs that were previously unrelated to that gene, resulting in ectopic gene repression. In addition, sequence alterations may modify the Gibbs free energy (ΔG) of the miRNA–mRNA interaction and influence RNA secondary structure, making binding sites more or less accessible to the miRNA-induced silencing complex (miRISC) [51].

The functional impact of these variants has already been described in several human diseases. A well-characterized immunological example involves a polymorphism in the 3′UTR of HLA-C that alters the binding of miR-148a, thereby modulating HLA-C expression levels. Variations affecting this interaction have been associated with altered susceptibility to both infectious and immune-mediated diseases, including improved control of HIV infection and an increased risk of Crohn’s disease [18]. Similar mechanisms have also been reported in other genes. For example, a point mutation in the 3′UTR of the *GFPT1* gene, for example, creates a novel miRNA binding site, resulting in a reduction in protein production and leading to congenital myasthenic syndrome [68]. In the *TGFB3* gene, a single-nucleotide substitution in the 3′UTR was identified in a patient with arrhythmogenic right ventricular cardiomyopathy and was shown to alter reporter gene expression in vitro [69]. Other examples include variants in the *RET* 3′UTR, which have been associated with altered mRNA secondary structure and clinical features of medullary thyroid carcinoma, with in silico analyses suggesting potential effects on mRNA degradation [66, 70], and polymorphisms in thymidylate synthase, which are linked to colorectal cancer prognosis [66, 71].

As previously described, the *MICA* gene is highly polymorphic, and this variability also extends to the 3′UTR region. In this context, the in silico study of specific interactions between *MICA* alleles and miRNAs becomes particularly relevant [51]. Different alleles exhibit variations in both the length and sequence of the 3′UTR; for instance, the MICA*008 allele contains a longer 3′UTR (327 nt) compared with other alleles (usually 170 nt) due to the premature stop-codon, thereby altering the potential repertoire of miRNA binding sites.

**Fig. 3:**
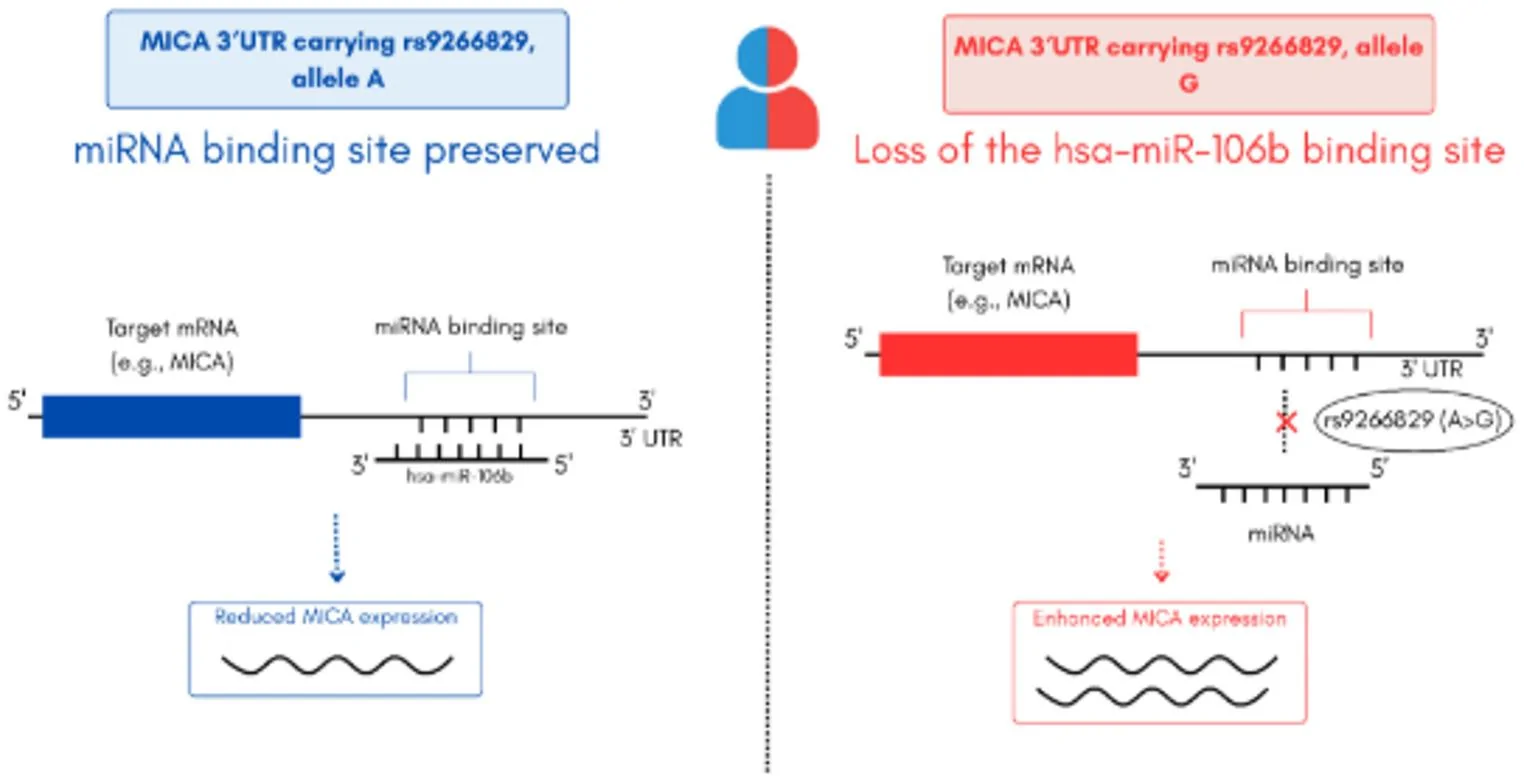
Allele-specific effects of SNPs on miRNA binding. Conceptual representation of the predicted impact of the MICA 3′UTR polymorphism rs9266829 (A > G) on miRNA-mediated regulation. In the presence of the rs9266829/A allele, hsa-miR-106b is predicted to efficiently bind the *MICA* 3′UTR, promoting mRNA degradation and/or translational repression. Conversely, the rs9266829/G variant disrupts this interaction, thereby increasing mRNA stability and potentially enhancing MICA expression. This schematic illustrates a proposed mechanism by which naturally occurring allelic variants may influence post-transcriptional regulation of MICA. Created by the authors using Canva and Microsoft PowerPoint

One example is the rs9266829 (A > G) polymorphism, for which in silico analyses predicted that the A allele preserves binding sites for several miRNAs, including members of the miR-17 ~ 92 family and related clusters, whereas the G allele disrupts some of these interactions. As a result, the G allele may be associated with reduced miRNA-mediated repression and potentially higher MICA expression (Fig. 3). Similar effects have also been predicted for other *MICA* 3′UTR polymorphisms, including rs1880, rs9266825, and rs9266826 [51]. Such variants provide a plausible molecular mechanism by which allele-specific sequence differences may modulate miRNA binding efficiency and ultimately influence MICA expression (Fig. 3).

Beyond computational predictions, studies characterizing the genetic diversity of the *MICA* 3′UTR have established that this region contains naturally occurring polymorphisms that may contribute to post-transcriptional regulation. Luo et al. (2013), in a sequencing-based analysis of 104 unrelated individuals, identified nine polymorphic sites in the *MICA* 3′UTR, which were organized into seven distinct *MICA* 3′UTR alleles and showed strong linkage disequilibrium. The authors highlighted that this previously uncharacterized genetic variation could be relevant to the mechanisms underlying MICA post-transcriptional regulation, although the functional consequences of these variants were not experimentally assessed [72].

Subsequent work further expanded the characterization of *MICA* 3′UTR diversity. Cox et al. (2018) identified 17 extended *MICA* haplotypes incorporating the coding region and 3′UTR in International Histocompatibility Workshop reference cell lines, including four previously unreported haplotypes [73]. Although the study did not directly evaluate miRNA binding or post-transcriptional activity, the authors emphasized that this polymorphic 3′UTR repertoire may have functional relevance and could provide a basis for future investigation of miRNA-mediated repression. These findings indicate that the sequence context potentially targeted by miRNAs is not invariant across *MICA* haplotypes, providing a genetic basis for the hypothesis that allele-specific differences in post-transcriptional regulation may occur.

An attempt to address this possibility was made by Tonnerre et al. (2013) in endothelial cells from kidney transplant donors [74]. The authors investigated whether the increased MICA expression associated with the A5.1 mutation could be explained by differences in miRNA-mediated regulation. Although they detected no quantitative differences in several miRNAs previously reported to regulate MICA, sequencing of the proximal 3′UTR identified seven novel SNPs at positions + 19, +24, + 28, +50, + 80, +122, and + 157 relative to the stop codon. None of these variants was specifically associated with the *MICA* A5.1 phenotype, and their functional effects on miRNA binding were not directly tested. Nevertheless, the authors explicitly considered the possibility that polymorphisms within the 3′UTR could impair miRNA binding and thereby contribute to allele-specific regulation of MICA expression. An overview of experimentally validated miRNAs targeting *MICA*, together with predicted effects of naturally occurring 3′UTR variants on miRNA interactions, is provided in Table 1.

Taken together, these studies establish that naturally occurring variation in the *MICA* 3′UTR is substantial, while its functional consequences for miRNA-mediated regulation remain incompletely characterized. Thus, although experimental studies using engineered mutations have demonstrated that alterations in *MICA* 3′UTR target sites can disrupt miRNA-mediated repression [60], direct experimental evidence linking naturally occurring *MICA* 3′UTR variants to allele-specific changes in miRNA binding and *MICA* expression remains limited. This distinction is particularly relevant because naturally occurring variants could alter miRNA–mRNA recognition without necessarily abolishing the interaction altogether, potentially resulting in quantitative rather than binary effects on post-transcriptional regulation.

Given that MICA surface expression provides an important activating signal for NK cells through the NKG2D receptor, allele-dependent differences in post-transcriptional regulation could influence the magnitude of MICA-mediated immune signaling. In this context, differential miRNA regulation represents a plausible mechanism through which naturally occurring *MICA* variants could contribute to variation in tumor–immune interactions and, potentially, immune evasion. However, this possibility remains to be directly tested. Overall, the available evidence highlights the value of allele-specific analyses of *MICA* for refining our understanding of how genetic variation may shape post-transcriptional regulation, immune response modulation, and disease susceptibility, and provides a rationale for investigating these mechanisms in the context of precision medicine.

**Table 1:** Experimentally validated and computationally predicted miRNA interactions with MICA

miRNA	Target	Cell type / model	Disease / condition	Allele / 3′UTR	Validation / prediction method	Functional readout	Evidence type	Reference
miR-93	*MICA*	HeLa, Hep3B, Huh7	Hepatocellular carcinoma	*MICA* 3′UTR; engineered seed-site mutations	Luciferase reporter; site-directed mutagenesis; miRNA manipulation; flow cytometry	MICA expression; reporter activity; NKG2D binding; NK-cell-mediated killing	Experimental	[60]
miR-106b	*MICA*	HeLa, Hep3B, Huh7	Hepatocellular carcinoma	*MICA* 3′UTR; engineered seed-site mutations	Luciferase reporter; site-directed mutagenesis; miRNA manipulation	MICA expression; reporter activity; NKG2D binding; NK-cell-mediated killing	Experimental	[60]
miR-25/93/106b cluster	*MICA*	Huh7, HLE, PLC/PRF/5, Hep3B	HCC/chronic hepatitis- associated liver cancer	*MICA* 3′UTR	Lentiviral transduction; flow cytometry; in vivo tumor-clearance assay	MICA expression; sMICA; NKG2D-ligand activity; NK-cell susceptibility	Experimental	[60]
miR-20a	*MICA*	HEK293T, SW480, HCT116	Colorectal cancer	*MICA* 3′UTR; wild-type and mutant reporter constructs	Luciferase reporter; qRT-PCR; flow cytometry; miRNA manipulation	MICA mRNA/protein; NK-cell cytotoxicity	Experimental	[61]
miR-93	*MICA*	-	-	rs9266829 A/G	In silico miRNA-binding prediction; minimum free energy (MFE) comparison	Predicted allele-dependent change in binding	In silico	[51]
miR-106a-5p	*MICA*	-	-	rs9266829 A/G	In silico prediction; MFE comparison	Predicted allele-dependent change in interaction energy	In silico	[51]
miR-106b	*MICA*	-	-	rs9266829 A/G	In silico prediction; MFE/secondary-structure analysis	Predicted loss of binding with G allele	In silico	[51]
miR-17-5p	*MICA*	-	-	rs9266829 A/G	In silico prediction; MFE comparison	Predicted allele-dependent change in interaction	In silico	[51]
miR-20a-5p	*MICA*	-	-	rs9266829 A/G	In silico prediction; MFE comparison	Predicted change in binding energy	In silico	[51]
miR-20b-5p	*MICA*	-	-	rs9266829 A/G	In silico prediction; MFE comparison	Predicted allele/UTR-dependent binding	In silico	[51]
miR-1207-5p	*MICA*	-	-	rs1880 C/A	In silico prediction; MFE comparison	Predicted allele-dependent binding	In silico	[51]
miR-711	*MICA*	-	-	rs9266825 C/A	In silico prediction; MFE comparison	Predicted gain/loss of interaction depending on UTR	In silico	[51]
miR-636	*MICA*	-	-	rs9266829 A/G	miRNASNP/in silico prediction	Predicted loss of binding with G allele	In silico	[51]
miR-758-3p	*MICA*	-	-	rs9266829 A/G	miRNASNP/in silico prediction	Predicted loss of binding with G allele	In silico	[51]
miR-581	*MICA*	-	-	rs9266829 A/G	miRNASNP/in silico prediction	Predicted loss of binding with G allele	In silico	[51]

## Where do we go from here: gaps in knowledge and future perspectives

Despite scientific advances and the identification of several miRNA-mediated regulatory mechanisms, important gaps still remain in understanding how specific allelic variants may influence miRNA biogenesis and their interactions with target genes, ultimately affecting complex post-transcriptional regulatory networks [67]. This aspect becomes particularly relevant in the context of the *MICA* gene. Since miRNAs regulate post-transcriptional gene expression through binding to target mRNAs, variations or polymorphisms within the *MICA* gene may alter these interactions. This suggests that allelic diversity may influence regulatory mechanisms associated with susceptibility to and progression of several pathological conditions [75, 76].

Recent studies have demonstrated that even small changes in mRNA sequence may significantly affect miRNA-mediated regulatory mechanisms. Despite this, most studies still investigate SNPs, gene expression, or miRNA regulation in isolation, and only a few integrate these distinct molecular processes. Given that alterations in any of these components may influence the others, an integrated understanding of genetic variability, miRNA–mRNA interactions, and gene expression is essential for elucidating the mechanisms underlying immune responses and their potential impacts on individuals [67, 76–78].

Several studies underscore the importance of bioinformatic predictions in investigating miRNA-mediated gene regulation. However, many results obtained through in silico approaches or preliminary models still require experimental validation to confirm their functional relevance. In this context, the integration of SNPs, mRNAs, and miRNAs has proven essential for understanding disease-associated mechanisms and may eventually contribute to the development of personalized therapies. Nevertheless, important challenges remain in experimentally confirming and validating their biological effects. Therefore, expanding experimental studies that support and complement computational analyses is essential [79, 80].

This mini-review highlights that understanding the key miRNAs regulating the *MICA* gene offers new perspectives across several fields of research, including infectious diseases, autoimmune disorders, pregnancy-related conditions, cancer, and even transplantation. This is particularly relevant because the MICA–NKG2D axis plays a fundamental role in maintaining the basal state of the immune system, directly influencing outcomes in these clinical contexts.

## Conclusions

Taken together, the evidence reviewed here establishes that miRNA-mediated regulation is an important component of MICA post-transcriptional control and that changes in MICA expression can influence NKG2D-mediated immune recognition. Experimental studies have demonstrated that several miRNAs directly regulate *MICA* through its 3′UTR, with consequences for MICA expression and NK-cell responses [60]. In parallel, genetic studies have established that the *MICA* 3′UTR contains naturally occurring polymorphisms and haplotypes, demonstrating that the regulatory sequence targeted by miRNAs is genetically variable among individuals [72–74].

Computational studies further suggest that naturally occurring *MICA* 3′UTR variants may alter miRNA-binding sites, interaction energies, or the repertoire of miRNAs potentially targeting different *MICA* alleles [51]. These findings provide a plausible mechanism by which genetic variation could contribute to allele-dependent differences in post-transcriptional regulation. However, an important distinction must be made between demonstrated miRNA regulation of *MICA*, experimentally characterized natural 3′UTR variation, and predicted effects of these variants on miRNA binding. Although experimental manipulation of *MICA* 3′UTR target sites has shown that changes in miRNA-responsive sequences can modify repression [60], this does not establish that naturally occurring *MICA* polymorphisms produce equivalent functional effects.

The major knowledge gap therefore lies in the lack of direct experimental evidence connecting a naturally occurring *MICA* 3′UTR variant to altered miRNA binding and, subsequently, to allele-specific changes in MICA expression and immune function. Such variants may not necessarily abolish miRNA recognition; instead, they may quantitatively modify binding affinity, target-site accessibility, or the relative contribution of individual miRNAs to *MICA* regulation. Addressing this possibility will require integrated allele-specific approaches combining naturally occurring *MICA* sequences or haplotypes, miRNA perturbation, direct assessment of miRNA–*MICA* interactions, gene and protein expression analyses, and functional evaluation of NKG2D-mediated immune responses.

Overall, the available evidence supports a model in which genetic variation, miRNA-mediated post-transcriptional regulation, and MICA-dependent immune surveillance are interconnected, but the causal relationship between naturally occurring *MICA* variants and allele-specific miRNA regulation remains to be established. Defining these relationships may provide new insights into inter-individual differences in immune responses and disease susceptibility and, ultimately, contribute to the identification of molecular biomarkers and more individualized strategies in precision medicine.

## Data Availability

No datasets were generated or analysed during the current study.

## Abbreviations

3′UTR: 3′ Untranslated region
5′UTR: 5′ Untranslated region
ADAM: A disintegrin and metalloproteinase
AGO: Argonaute protein
CDS: Coding sequence
DNA: Deoxyribonucleic acid
DSA: Donor-specific antibodies
ERp5: Endoplasmic reticulum protein 5
GPI: Glycosylphosphatidylinositol
HIV: Human immunodeficiency virus
HLA-B: Human leukocyte antigen B
HLA-C: Human leukocyte antigen C
MHC: Major histocompatibility complex
MICA: MHC class I chain-related chain A
MICB: MHC class I chain-related protein B
MICC: MHC class I chain-related protein C
MICD: MHC class I chain-related protein D
MICE: MHC class I chain-related protein E
MICF: MHC class I chain-related protein F
MICG: MHC class I chain-related protein G
miRISC: MicroRNA-induced silencing complex
miRNA: MicroRNA
MMPs: Matrix metalloproteinases
MRE: MicroRNA response element
mRNA: Messenger RNA
MFE: Minimum free energy
NKG2D: Natural killer cell group 2-D
pre-miRNA: Precursor microRNA
pri-miRNA: Primary microRNA
RBP: RNA-binding protein
RNA: Ribonucleic acid
RNase III: Ribonuclease III
SLE: Systemic lupus erythematosus
sMICA: Soluble MICA
SNP: Single nucleotide polymorphism
STR: Short tandem repeat

## Declarations

### Generative AI Use

Generative artificial intelligence (AI) tools were used exclusively to support language revision, text refinement, and readability improvement during manuscript preparation. Specifically, Grammarly, ChatGPT (OpenAI) and NotebookLM (Google) were used for assistance with grammar correction, sentence restructuring, and language polishing. The authors critically reviewed, edited, and validated all generated content and remain fully responsible for the accuracy, originality, and integrity of the manuscript.

## References

[1] Beňačka R, Szabóová D, Guľašová Z et al (2023) Non-Coding RNAs in Human Cancer and Other Diseases: Overview of the Diagnostic Potential. Int J Mol Sci 24:16213. 10.3390/ijms242216213 PMC10671391 38003403

[2] Wightman B, Ha I, Ruvkun G (1993) Posttranscriptional regulation of the heterochronic gene lin-14 by lin-4 mediates temporal pattern formation in C. elegans. Cell 75:855–862. 10.1016/0092-8674(93)90530-4 8252622

[3] Lee RC, Feinbaum RL, Ambros V (1993) The C. elegans heterochronic gene lin-4 encodes small RNAs with antisense complementarity to lin-14. Cell 75:843–854. 10.1016/0092-8674(93)90529-y 8252621

[4] Kozomara A, Birgaoanu M, Griffiths-Jones S (2019) miRBase: from microRNA sequences to function. Nucleic Acids Res 47:D155–D162. 10.1093/nar/gky1141 PMC6323917 30423142

[5] Kehl T, Backes C, Kern F et al (2017) About miRNAs, miRNA seeds, target genes and target pathways. Oncotarget 8:107167–107175. 10.18632/oncotarget.22363 PMC5739805 29291020

[6] Grimson A, Farh KK-H, Johnston WK et al (2007) MicroRNA Targeting Specificity in Mammals: determinants beyond Seed Pairing. Mol Cell 27:91–105. 10.1016/j.molcel.2007.06.017 PMC3800283 17612493

[7] O’Brien J, Hayder H, Zayed Y, Peng C (2018) Overview of MicroRNA Biogenesis, Mechanisms of Actions, and Circulation. Front Endocrinol 9:402. 10.3389/fendo.2018.00402 PMC6085463 30123182

[8] Mayr C (2019) What Are 3′ UTRs Doing? Cold Spring Harb Perspect Biol. 11:a034728. 10.1101/cshperspect.a034728 PMC6771366 30181377

[9] Ebert MS, Sharp PA (2012) Roles for microRNAs in conferring robustness to biological processes. Cell 149:515–524. 10.1016/j.cell.2012.04.005 PMC3351105 22541426

[10] Gu S, Jin L, Zhang F et al (2009) Biological basis for restriction of microRNA targets to the 3′ untranslated region in mammalian mRNAs. Nat Struct Mol Biol 16:144–150. 10.1038/nsmb.1552 PMC2713750 19182800

[11] Sarkar N, Kumar A (2025) Paradigm shift: microRNAs interact with target gene promoters to cause transcriptional gene activation or silencing. Exp Cell Res 444:114372. 10.1016/j.yexcr.2024.114372 39662662

[12] Ilker E, Hinczewski M (2024) Bioenergetic costs and the evolution of noise regulation by microRNAs. Proc Natl Acad Sci 121:e2308796121. 10.1073/pnas.2308796121 PMC10907262 38386708

[13] Detzer A, Engel C, Wünsche W, Sczakiel G (2011) Cell stress is related to re-localization of Argonaute 2 and to decreased RNA interference in human cells. Nucleic Acids Res 39:2727–2741. 10.1093/nar/gkq1216 PMC3074141 21148147

[14] Chen X, Ba Y, Ma L et al (2008) Characterization of microRNAs in serum: a novel class of biomarkers for diagnosis of cancer and other diseases. Cell Res 18:997–1006. 10.1038/cr.2008.282 18766170

[15] Herbst F, Lang TJL, Eckert ESP et al (2021) The balance between the intronic miR-342 and its host gene Evl determines hematopoietic cell fate decision. Leukemia 35:2948–2963. 10.1038/s41375-021-01267-5 PMC8478659 34021250

[16] Koralov SB, Muljo SA, Galler GR et al (2008) Dicer ablation affects antibody diversity and cell survival in the B lymphocyte lineage. Cell 132:860–874. 10.1016/j.cell.2008.02.020 18329371

[17] Wu C-J, Lu L-F (2017) MicroRNA in Immune Regulation. In: Current topics in microbiology and immunology. pp 249–267 10.1007/82_2017_65 PMC8476048 28900683

[18] Kulkarni S, Qi Y, O’hUigin C et al (2013) Genetic interplay between HLA-C and MIR148A in HIV control and Crohn disease. Proc Natl Acad Sci U S A 110:20705–20710. 10.1073/pnas.1312237110 PMC3870724 24248364

[19] Bahram S, Bresnahan M, Geraghty DE, Spies T (1994) A second lineage of mammalian major histocompatibility complex class I genes. Proc Natl Acad Sci U S A 91:6259–6263. 10.1073/pnas.91.14.6259 PMC44180 8022771

[20] Choy M-K, Phipps ME (2010) MICA polymorphism: biology and importance in immunity and disease. Trends Mol Med 16:97–106. 10.1016/j.molmed.2010.01.002 20153697

[21] Bahram S (2000) MIC genes: from genetics to biology. Adv Immunol 76:1–60. 10.1016/s0065-2776(01)76018-x 11079097

[22] Cruvinel W, de Mesquita Júnior M, Araújo D JAP, et al (2010) Sistema imunitário: Parte I. Fundamentos da imunidade inata com ênfase nos mecanismos moleculares e celulares da resposta inflamatória. Rev Bras Reumatol 50:434–447. 10.1590/S0482-50042010000400008

[23] Vavilov MN, Suslova TA, Burmistrova AL (2022) Frequency and parameters of linkage disequilibrium of the two-locus HLA-B~MICA haplotypes in russians from chelyabinsk region. Russ J Immunol 25:139–146. 10.46235/1028-7221-1134-FAP

[24] Raulet DH, Gasser S, Gowen BG et al (2013) Regulation of Ligands for the NKG2D Activating Receptor. Annu Rev Immunol 31:413–441. 10.1146/annurev-immunol-032712-095951 PMC4244079 23298206

[25] Vulpis E, Loconte L, Peri A et al (2022) Impact on NK cell functions of acute versus chronic exposure to extracellular vesicle-associated MICA: Dual role in cancer immunosurveillance. J Extracell Vesicles 11:e12176. 10.1002/jev2.12176 PMC8720178 34973063

[26] Yurevna SA, Smirnov IV, Samoylovich MP (2022) Stress-induced MICA and MICB molecules in oncology. Med Immunol Russ 24:433–454. 10.15789/1563-0625-SIM-2480

[27] Zhang J, Luo Q, Li X et al (2023) Novel role of immune-related non-coding RNAs as potential biomarkers regulating tumour immunoresponse via MICA/NKG2D pathway. Biomark Res 11:86. 10.1186/s40364-023-00530-4 PMC10546648 37784183

[28] Zwirner NW, Dole K, Stastny P (1999) Differential surface expression of MICA by endothelial cells, fibroblasts, keratinocytes, and monocytes. Hum Immunol 60:323–330. 10.1016/s0198-8859(98)00128-1 10363723

[29] Groh V, Bahram S, Bauer S et al (1996) Cell stress-regulated human major histocompatibility complex class I gene expressed in gastrointestinal epithelium. Proc Natl Acad Sci U S A 93:12445–12450. 10.1073/pnas.93.22.12445 PMC38011 8901601

[30] Waldhauer I, Goehlsdorf D, Gieseke F et al (2008) Tumor-associated MICA is shed by ADAM proteases. Cancer Res 68:6368–6376. 10.1158/0008-5472.CAN-07-6768 18676862

[31] Wang K, Xuan Z, Liu X et al (2022) Immunomodulatory role of metalloproteinase ADAM17 in tumor development. Front Immunol 13:1059376. 10.3389/fimmu.2022.1059376 PMC9715963 36466812

[32] Wang X, Lundgren AD, Singh P et al (2009) An six-amino acid motif in the alpha3 domain of MICA is the cancer therapeutic target to inhibit shedding. Biochem Biophys Res Commun 387:476–481. 10.1016/j.bbrc.2009.07.062 PMC2737406 19615970

[33] Xing S, Ferrari de Andrade L (2020) NKG2D and MICA/B shedding: a tag game between NK cells and malignant cells. Clin Transl Immunol 9:e1230. 10.1002/cti2.1230 PMC7754731 33363734

[34] Du C, Bevers J, Cook R et al (2019) MICA immune complex formed with alpha 3 domain-specific antibody activates human NK cells in a Fc-dependent manner. J Immunother Cancer 7:207. 10.1186/s40425-019-0687-9 PMC6685158 31387641

[35] Fuertes MB, Domaica CI, Zwirner NW (2021) Leveraging NKG2D Ligands in Immuno-Oncology. Front Immunol 12:713158. 10.3389/fimmu.2021.713158 PMC8358801 34394116

[36] Molfetta R, Quatrini L, Zitti B et al (2016) Regulation of NKG2D Expression and Signaling by Endocytosis. Trends Immunol 37:790–802. 10.1016/j.it.2016.08.015 27667711

[37] Pachani P, Godbole RR, Kshersagar J et al (2022) A comparative study of smica in various body fluids of diagnosed cervical cancer patients and healthy women. Obstet Gynecol Sci 65:37–45. 10.5468/ogs.21121 PMC8784934 34743476

[38] Wang S, Onyeaghala GC, Pankratz N et al (2023) Associations between MICA and MICB Genetic Variants, Protein Levels, and Colorectal Cancer: Atherosclerosis Risk in Communities (ARIC). Cancer Epidemiol Biomarkers Prev 32:784–794. 10.1158/1055-9965.EPI-22-1113 PMC10239349 36958849

[39] Kshersagar J, Damle MN, Bedge P et al (2022) Downregulation of MICA/B tumor surface expressions and augmented soluble MICA serum levels correlate with disease stage in breast cancer. Breast Dis 41:471–480. 10.3233/BD-220023 36641654

[40] Gutiérrez-Bautista JF, Martinez-Chamorro A, Rodriguez-Nicolas A et al (2022) Major Histocompatibility Complex Class I Chain-Related α (MICA) STR Polymorphisms in COVID-19 Patients. Int J Mol Sci 23:6979. 10.3390/ijms23136979 PMC9266713 35805975

[41] Gambelunghe G, Brozzetti AL, Ghaderi M et al (2006) MICA A8: a new allele within MHC class I chain-related A transmembrane region with eight GCT repeats. Hum Immunol 67:1005–1007. 10.1016/j.humimm.2006.10.007 17174750

[42] Mizuki N, Ota M, Kimura M et al (1997) Triplet repeat polymorphism in the transmembrane region of the MICA gene: A strong association of six GCT repetitions with Behçet disease. Proc Natl Acad Sci 94:1298–1303. 10.1073/pnas.94.4.1298 PMC19785 9037047

[43] Tani R, Ito N, Matsui K et al (2021) MICA A5.1 homozygous genotype is associated with a risk for early-onset oral cancer. Oral Oncol 116:105256. 10.1016/j.oraloncology.2021.105256 33730629

[44] Zou Y, Han M, Wang Z, Stastny P (2006) MICA allele-level typing by sequence-based typing with computerized assignment of polymorphic sites and short tandem repeats within the transmembrane region. Hum Immunol 67:145–151. 10.1016/j.humimm.2006.02.016 16698436

[45] Ashiru O, López-Cobo S, Fernández-Messina L et al (2013) A GPI anchor explains the unique biological features of the common NKG2D-ligand allele MICA*008. Biochem J 454:295–302. 10.1042/BJ20130194 23772752

[46] Rodrigues AM (2022) Polimorfismos em éxons do gene MICA e o impacto funcional dessas variantes em diferentes regiões biogeográficas. Universidade Estadual Paulista (Unesp). Undergraduate thesis

[47] Souza CF, Noguti EN, Visentainer JEL et al (2012) HLA and MICA genes in patients with tuberculosis in Brazil. Tissue Antigens 79:58–63. 10.1111/j.1399-0039.2011.01789.x 22032421

[48] Yamakawa RH, Saito PK, Gelmini GF et al (2017) MICA diversity and linkage disequilibrium with HLA-B alleles in renal-transplant candidates in southern Brazil. PLoS ONE 12:e0176072. 10.1371/journal.pone.0176072 PMC5395226 28419176

[49] Albrecht V, Paech C, Putke K et al (2026) Advances in MICA genotyping: characterization of 406 novel alleles and their frequencies in multiple populations. Front Immunol 17:1741611. 10.3389/fimmu.2026.1741611 PMC12989365 41846934

[50] Luo Q, Yin X, Zhu Q et al (2025) Two major human phenotypes of MICA molecules and their differential activation to NK cells via NKG2D receptor. Front Immunol 16:1563872. 10.3389/fimmu.2025.1563872 PMC12127157 40458406

[51] Toledo-Stuardo K, Ribeiro CH, Campos I et al (2023) Impact of MICA 3′UTR allelic variability on miRNA binding prediction, a bioinformatic approach. Front Genet 14:1273296. 10.3389/fgene.2023.1273296 PMC10749337 38146340

[52] Machuldova A, Holubova M, Caputo VS et al (2021) Role of Polymorphisms of NKG2D Receptor and Its Ligands in Acute Myeloid Leukemia and Human Stem Cell Transplantation. Front Immunol 12:651751. 10.3389/fimmu.2021.651751 PMC8044845 33868289

[53] Wang C-M, Tan K-P, Wu Y-JJ et al (2024) Functional MICA Variants Are Differentially Associated with Immune-Mediated Inflammatory Diseases. Int J Mol Sci 25:3036. 10.3390/ijms25053036 PMC10931785 38474281

[54] Ciriaco VA, de O, Rodrigues AM, da Silva Tibúrcio BC et al (2024) The *MICA* deletion across different populations. Hum Immunol 85:111183. 10.1016/j.humimm.2024.111183 39571451

[55] Isernhagen A, Malzahn D, Viktorova E et al (2015) The MICA-129 dimorphism affects NKG2D signaling and outcome of hematopoietic stem cell transplantation. EMBO Mol Med 7:1480–1502. 10.15252/emmm.201505246 PMC4644379 26483398

[56] Yadav D, Ngolab J, Lim RS-H et al (2009) Cutting edge: down-regulation of MHC class I-related chain A on tumor cells by IFN-gamma-induced microRNA. J Immunol 182:39–43. 10.4049/jimmunol.182.1.39 PMC2714222 19109132

[57] Vaxevanis C, Bachmann M, Seliger B (2024) Immune modulatory microRNAs in tumors, their clinical relevance in diagnosis and therapy. J Immunother Cancer 12:e009774. 10.1136/jitc-2024-009774 PMC11367391 39209767

[58] Jasinski-Bergner S, Mandelboim O, Seliger B (2020) Molecular mechanisms of human herpes viruses inferring with host immune surveillance. J Immunother Cancer 8:e000841. 10.1136/jitc-2020-000841 PMC7333871 32616556

[59] Stern-Ginossar N, Gur C, Biton M et al (2008) Human microRNAs regulate stress-induced immune responses mediated by the receptor NKG2D. Nat Immunol 9:1065–1073. 10.1038/ni.1642 18677316

[60] Kishikawa T, Otsuka M, Yoshikawa T et al (2013) Regulation of the expression of the liver cancer susceptibility gene MICA by microRNAs. Sci Rep 3:2739. 10.1038/srep02739 PMC3781398 24061441

[61] Tang S, Fu H, Xu Q, Zhou Y (2019) miR-20a regulates sensitivity of colorectal cancer cells to NK cells by targeting MICA. Biosci Rep 39:BSR20180695. 10.1042/BSR20180695 PMC6603280 30988067

[62] Pesce S, Greppi M, Ferretti E et al (2020) miRNAs in NK Cell-Based Immune Responses and Cancer Immunotherapy. Front Cell Dev Biol 8:119. 10.3389/fcell.2020.00119 PMC7053181 32161759

[63] Wong T-S, Chen S, Zhang M-J et al (2018) Epstein–Barr virus–encoded microRNA BART7 downregulates major histocompatibility complex class I chain–related peptide A and reduces the cytotoxicity of natural killer cells to nasopharyngeal carcinoma. Oncol Lett 16:2887–2892. 10.3892/ol.2018.9041 PMC6096257 30127876

[64] Xie J, Liu M, Li Y et al (2014) Ovarian tumor-associated microRNA-20a decreases natural killer cell cytotoxicity by downregulating MICA/B expression. Cell Mol Immunol 11:495–502. 10.1038/cmi.2014.30 PMC4197204 24813230

[65] Breunig C, Pahl J, Küblbeck M et al (2017) MicroRNA-519a-3p mediates apoptosis resistance in breast cancer cells and their escape from recognition by natural killer cells. Cell Death Dis 8:e2973. 10.1038/cddis.2017.364 PMC5596553 28771222

[66] Su J-Y, Wang Y-L, Hsieh Y-T et al (2025) Multiplexed assays of human disease-relevant mutations reveal UTR dinucleotide composition as a major determinant of RNA stability. eLife 13:RP97682. 10.7554/eLife.97682 PMC11835390 39964837

[67] Komatsu S, Kitai H, Suzuki HI (2023) Network Regulation of microRNA Biogenesis and Target Interaction. Cells 12:306. 10.3390/cells12020306 PMC9856966 36672241

[68] Dusl M, Senderek J, Müller JS et al (2015) A 3′-UTR mutation creates a microRNA target site in the GFPT1 gene of patients with congenital myasthenic syndrome. Hum Mol Genet 24:3418–3426. 10.1093/hmg/ddv090 25765662

[69] Beffagna G, Occhi G, Nava A et al (2005) Regulatory mutations in transforming growth factor-beta3 gene cause arrhythmogenic right ventricular cardiomyopathy type 1. Cardiovasc Res 65:366–373. 10.1016/j.cardiores.2004.10.005 15639475

[70] Ceolin L, Romitti M, Siqueira DR et al (2016) Effect of 3’UTR RET Variants on RET mRNA Secondary Structure and Disease Presentation in Medullary Thyroid Carcinoma. PLoS ONE 11:e0147840. 10.1371/journal.pone.0147840 PMC4734678 26829565

[71] Jeon Y-J, Cho S-H, Kim E-J et al (2021) 3’-UTR Polymorphisms in Thymidylate Synthase with Colorectal Cancer Prevalence and Prognosis. J Pers Med 11:537. 10.3390/jpm11060537 PMC8228787 34207922

[72] Luo J, Tian W, Liu XX et al (2013) The genetic architecture of 3’untranslated region of the MICA gene: polymorphisms and haplotypes. Hum Immunol 74:1321–1325. 10.1016/j.humimm.2013.06.024 23806266

[73] Cox ST, Hernandez D, Danby R et al (2018) Diversity and characterisation of polymorphic 3’ untranslated region haplotypes of MICA and MICB genes. HLA 92:392–402. 10.1111/tan.13434 30471210

[74] Tonnerre P, Gérard N, Chatelais M et al (2013) MICA Variant Promotes Allosensitization after Kidney Transplantation. J Am Soc Nephrol 24:954–966. 10.1681/ASN.2012080814 PMC3665393 23539759

[75] Levantini E, Rizzo M (2024) miRNAs: From Master Regulators of Gene Expression to Biomarkers Involved in Intercellular Communication. Biomedicines 12:721. 10.3390/biomedicines12040721 PMC11048632 38672077

[76] Orbán TI (2023) One locus, several functional RNAs-emerging roles of the mechanisms responsible for the sequence variability of microRNAs. Biol Futura 74:17–28. 10.1007/s42977-023-00154-7 36847925

[77] Nalbant E, Akkaya-Ulum YZ (2024) Exploring regulatory mechanisms on miRNAs and their implications in inflammation-related diseases. Clin Exp Med 24:142. 10.1007/s10238-024-01334-y PMC11222192 38958690

[78] Wilk G, Braun R (2018) regQTLs: Single nucleotide polymorphisms that modulate microRNA regulation of gene expression in tumors. PLoS Genet 14:e1007837. 10.1371/journal.pgen.1007837 PMC6343932 30557297

[79] Diener C, Keller A, Meese E (2022) Emerging concepts of miRNA therapeutics: from cells to clinic. Trends Genet TIG 38:613–626. 10.1016/j.tig.2022.02.006 35303998

[80] Seyhan AA (2024) Trials and Tribulations of MicroRNA Therapeutics. Int J Mol Sci 25:1469. 10.3390/ijms25031469 PMC10855871 38338746

